# Experience with the use of a digital sleep diary in symptom management by individuals with insomnia -a pilot mixed method study

**DOI:** 10.1016/j.sleepx.2023.100093

**Published:** 2023-11-14

**Authors:** Thea Christine Thorshov, Caroline Tonje Øverby, Diana Dobran Hansen, Way Kiat Bong, Knut Skifjeld, Petter Hurlen, Toril Dammen, Anne Moen, Harald Hrubos-Strøm

**Affiliations:** a Division of Surgery, Department of Otorhinolaryngology, Akershus University Hospital, Lørenskog, Norway; b Faculty of Technology, Art and Design, Department of Computer Science, Human-Computer Interaction and Universal Design of ICT, Oslo Metropolitan University, Oslo, Norway; c Norsk e-helse, Oslo, Norway; d Division of Clinical Informatics, Department of Diagnostics and Technology, Akershus University Hospital, Lørenskog, Norway; e Faculty of Medicine, Institute of Clinical Medicine, Campus Ahus, University of Oslo, Norway; f Faculty of Medicine, Institute of Health and Society, Department of Nursing Science, University of Oslo, Norway; g Faculty of Medicine, Institute of Clinical Medicine, University of Oslo, Norway; h Department of Research and Innovation, Division of Mental Health and Addiction, Oslo University Hospital, Oslo, Norway

**Keywords:** Insomnia, Patient-reported outcomes, Self-management, Motivation, Engagement, Feasibility, Digital sleep diary

## Abstract

**Background:**

Insomnia is the most common sleep disorder. The recommended treatment is cognitive behavioural therapy for insomnia (CBTi). A sleep diary is a core tool in CBTi. We have developed a digital sleep diary with a standardised feedback function.

**Aim:**

To study feasibility of the digital sleep diary in participants of the Akershus Sleep Apnea (ASAP) cohorts with difficulties falling asleep or maintaining sleep. To describe sleep diary engagement and explore experiences with the digital sleep diary with potential influences in insomnia symptom management.

**Material and methods:**

Twenty participants were recruited from the ASAP. All filled out a digital sleep diary up to 12 weeks. Treatment options provided were a self-help book (N = 11) or electroencephalography neurofeedback (N = 9) in addition to the sleep diary standardised feedback function. We collected quantitative data from the sleep diary reports and we sub-divided insomnia by sleep onset insomnia and non-sleep onset insomnia. Finally, we performed qualitative interviews.

**Results:**

The median number of entries to the sleep diary was 81 (25th quartile: 26, 75th quartile 84). In the qualitative analysis, we identified two main themes; “structure and overview” and “usability and digital features”.

**Conclusion:**

The sleep diary was found to be feasible when distributed in combination with a self-help book or electroencephalography neurofeedback. The qualitative results emphasised the importance of a timely graphical overview and visualisations of self-recorded sleep.

## Highlights

• Insomnia is the most common sleep disorder, but best treatment is not available.
• Digital sleep diary as a self-management tool for insomnia patients.
• Making effective insomnia treatment more available through new technology.
• Improving condition of insomnia patients through self-reporting digital sleep diary.

## 1 Introduction

Approximately 30 % of the population worldwide suffer from insomnia symptoms [1]. Symptoms of insomnia can impact the performance of everyday tasks and the declining quality of life [2]. Diagnostic criteria of chronic insomnia are defined by the International classification of sleep disorders, version 3 (ICSD-3) [3]. The main ICSD-3 criteria for chronic insomnia are 1: symptoms must be clinically significant and 2: that they persist for at least three nights a week over a three-month period [3]. Chronic insomnia affects 6–10 % of the adult population and can either be an independent disorder or comorbid with other conditions, such as depression, anxiety or obstructive sleep apnea (OSA) [[4], [5], [6]]. The co-occurrence of insomnia and OSA results in additive impairments to patients' sleep quality and quality of life. The most effective treatment of OSA is continuous positive airway pressure (CPAP), where patients are required to wear nasal or oro-nasal masks throughout the night [6]. However, adherence to CPAP has been shown to vary between patients with middle insomnia (difficulties maintaining sleep) and patients with sleep onset insomnia [7]. Insomnia symptoms and chronic insomnia represent a high financial and medical burden in Western countries, either through medication, psychotherapeutic treatment, through sick leave or early retirement [8].

Patients with chronic insomnia may be characterised by difficulties falling asleep (sleep onset insomnia) or problems maintaining sleep (non-sleep onset insomnia) [9]. The recommended treatment for both subtypes is lifestyle changes and cognitive behavioural therapy for insomnia (CBTi) [8]. Core elements of CBTi are sleep hygiene, stimulus control and sleep restriction [10,11]. However, traditional face-to-face CBTi is seldom offered in clinical practice. Reasons for this may be lack of resources and time [12]. The effect of CBTi has been reported to differ between subtypes of insomnia [5].

The primary tool in CBTi is a sleep diary. The *Consensus Sleep Diary* was developed by Carney and co-workers [13] and is still the golden standard for subjective sleep assessment. A digital sleep diary is a self-management tool that aims to gather information about the daily sleep pattern as a starting point for communication. Feedback has been based on manual calculations by healthcare providers or patients themselves. Patient-reported outcomes measurements (PROMs), such as a sleep diary, play an important role in health professionals’ ability to observe and understand the significance of sleep impairment [14]. Feedback from validated PROM systems provide information that advances clinical communication between patients and healthcare providers [15] and may also help patients to be more actively involved in their clinical evaluation and treatment [2,16]. A sleep diary, in combination with other elements of CBTi, aims to help individuals to reduce and manage insomnia symptoms [17].

Digital sleep diaries may replace traditional sleep diaries on paper as it provides advantages such as improving accuracy, providing immediate feedback and avoiding patients retrospectively reporting several days at a time [18]. In a systematic review of the clinical implementation and evaluation of mobile health applications for sleep disturbance, [19,20] identified 15 relevant papers, including eight individual applications. Of these, only four aimed to deliver a CBTi intervention, while six only studied the sleep diary feature. We have identified three recent studies validating a digital sleep diary [[19], [20], [21],^22^], but only one of these presented qualitative data [19,20]. Qualitative studies provide important and valuable insight on how people with insomnia utilise and experience a digital self-management tool and contribute to understanding patient perspectives [23]. In this study, we have developed a digital sleep diary based on *The Consensus Sleep Diary* with automatic, standardised feedback in the CAPABLE platform together with the company Norwegian e-health.

On this background, we aimed to study the sleep diary as a feasible digital self-management tool and describe engagement in participants of the Akershus Sleep Apnea (ASAP) cohorts with difficulties falling asleep or maintaining sleep. Moreover, we aimed to explore experiences with the digital sleep diary and impact on symptom management of insomnia.

## 2 Material and methods

### 2.1 Material/participants and procedure

Twenty-seven participants were invited from the ASAP epidemiological (n = 11) and clinical cohorts (n = 9) to participate in this pilot study. Participants with insomnia symptoms were invited to use a digital sleep diary and to receive additional, non-pharmacological treatment for insomnia described below. A diagnosis of insomnia was obtained by a modified DUKE structured interview for sleep disorders when invited to participate [24]. Sleep onset insomnia was defined when participants reported problems with sleep initiation in the DUKE interview. Participants not reporting sleep initiation problems were classified as non-sleep onset insomnia.

**Fig. 1:**
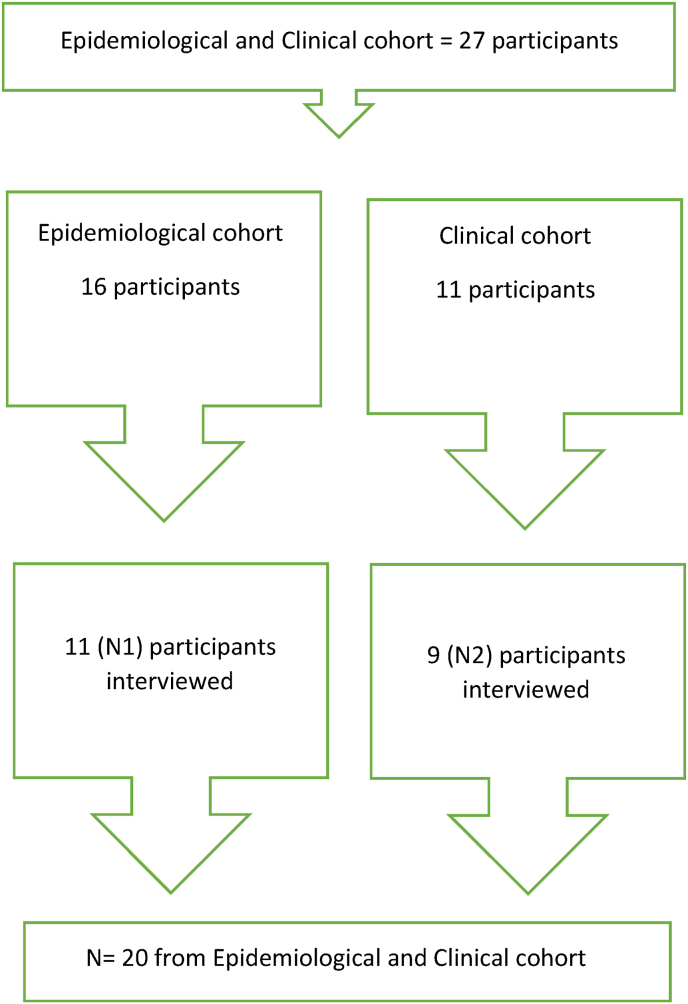
Flowchart.

The DUKE interview was conducted in October 2020 by phone in the epidemiological cohort, seven months after participating in the ASAP II study. The clinical cohort was interviewed in June of 2021, also seven months after participating in the study. In the epidemiological cohort [25], 16 participants consented to participate. Eleven of these participated in the qualitative study, as five participants did not respond to our interview appointment. In the clinical cohort, eleven participants accepted the invitation [26]. Nine participants from this cohort were interviewed, and two were out of reach. See flowchart, Fig. 1. Since both study cohorts were recruited from a follow-up examination conducted as part of the cohort studies, the only exclusion criterion for this study was age >80. Both cohorts were asked to complete the digital sleep diary for up to 12 weeks.

### 2.2 Interventions

**Fig. 2:**
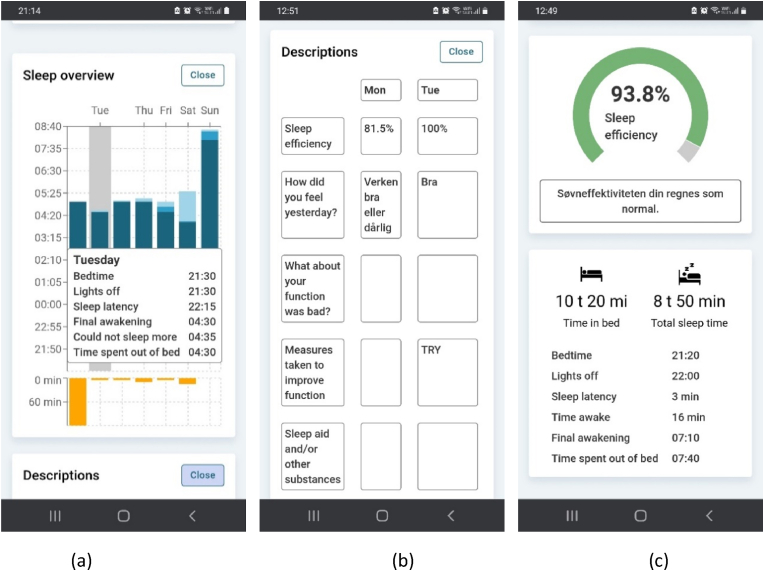
Standardised feedback from the sleep diary; 2 (a) histogram presenting an overview of sleep, 2 (b) table of text entered, 2 (c) sleep efficiency in percentage coupled with a respective description and suggestions to improve the sleep efficiency if required. Also, an average calculation of time in bed and total sleep time, bedtime, lights-off time, sleep latency in minutes, time awake in minute, time of final awakening and time spent out of bed, based on 3–7 entries during one week are presented.

The digital sleep diary in the CAPABLE platform is a digital self-management tool designed to provide standardised feedback about subjective sleep based on formulas proposed by Reed and Sacco [27] if completed at least three times per week (Fig. 2) [28]. Graphical feedback highlights subjective sleep efficiency (SSE), time in bed (TIB) and sleep onset latency (SOL). The diary differs from the Consensus Sleep Diary by collecting data once daily in the morning. Moreover, the digital sleep diary sends a daily reminder via SMS to record in the diary. The treatment options provided were a self-help book or electroencephalography neurofeedback in addition to the sleep diary standardised feedback function. The self-help book [29] about sleep was given to participants of the epidemiological cohort (n = 11). Participants of the clinical cohort (n = 9) received an intervention with electroencephalogram (EEG) neurofeedback developed by the company Drowzee AS (Oslo, Norway) (see supplement guide for description of the EEG device).

### 2.3 Design - mixed method

This study has a mixed-method design. Quantitative data summarises demographical data and data on subjective sleep measures. Moreover, the qualitative data explore experiences, describe engagement in the digital sleep diary, and potentially influences insomnia management.

### 2.4 Quantitative data and analysis

*The objective sleep parameters* studied were the Apnoea Hypopnea Index (AHI) and the objective sleep efficiency (OSE) measured at baseline in conjunction with the consultation in the data collection in the respective studies. Sleep was registered with a Nox Medical Self-Applied Somnography device (epidemiological cohort) and a Nox Medical A1 polysomnography (PSG) (clinical cohort). Manual scoring was performed using the latest scoring rules by the AASM Manual for the scoring of sleep and associated events (version 2.6, 2020). The scoring was done by an expert sleep technologist at Reykjavik University Sleep Institute, using Noxturnal Research (version 6.1.0.30257). See supplemental material for details.

*Subjective Sleep efficiency* from the sleep diary (SSE) was calculated based on duration of sleep episode (DSE) and time in bed (TIB) [27]. *Subjective sleep quality* (SSQ) was obtained from a question in the sleep diary asking, “Evaluate your sleep quality”.

Participants were divided into subgroups with sleep onset insomnia and non-sleep onset insomnia (sleep maintenance problems and early morning awakenings) based on the modified DUKE questionnaire at baseline [24]. *Engagement* in the digital sleep diary was measured by the mean number of entries in the digital sleep diary. The variable was categorised by a median split on 81 entries. One participant never logged in to the sleep diary, and the sum of participants was therefore 19 regarding this variable.

Statistical analysis was performed using STATA, Texas, United States of America. We used the Shapiro-Wilk Test to test for normality. Because only age was normally distributed, all variables are presented as quartiles and medians.

### 2.5 Qualitative data and analysis

The qualitative data were collected through individual interviews using a semi-structured interview guide. The questions were developed by experts in the field, based on extensive clinical and research experience. The questions seek to elicit experiences with the sleep diary; motivation for use, facilitation for behaviour change, practical application. Examples of interview questions include, “How is it to fill out the sleep diary?”, “How has the sleep diary contributed to your sleep behaviour?”, “What do you think of the report you received based on the sleep diary you had registered?” and “What are the reasons for you completing/not completing the sleep diary?”.

Interviews were conducted by phone (during COVID-19) by the first author. The Epidemiological cohort was interviewed during 2020, and the Clinical cohort was interviewed during 2021. All interviews were recorded with a field recorder and later imported to Services for Sensitive Data (Norwegian “Tjenester for Sensitive Data” (TSD)) at the University of Oslo. The interviews were transcribed in TSD. The interviews conducted with participants of the Epidemiological cohort lasted on average 21 min, whereas the average time was 30 min in the Clinical cohort. The transcribed material was a total of 230 pages (A4, 1.5 line spacing). The data was deidentified before being imported to Nvivo 12 software for analysis. We performed a thematic analysis using the six-stage method for thematic analysis [30]. First we coded the data in nodes, and then nodes were grouped into different subcategories and abstracted into main categories and themes. To ensure the rigor of the analysis, the authors TCT, WKB and AM read and discussed all transcripts to consolidate and agree upon the final version of the analysis. We adhered to the 15-point checklist of criteria for good thematic analysis to ensure the quality of our study [30].

### 2.6 Ethical considerations

The study is approved by the Regional Committees for Medical and Health Research Ethics (REK) and the data protection officer at the hospital. ASAP epidemiological cohort REK reference: 2017/2161 and data protection officer reference number: 2019_66. ASAP clinical cohort REK reference number: 50804 and data protection officer reference number: 2020_055. All participants were informed about the study and signed the consent form. The participants are deidentified, both in the quantitative and in the qualitative data.

## 3 Results

### 3.1 Quantitative results

Nine females and eleven males were included in this study. Characteristics of the participants are presented in Table 1. The median number of entries in the digital sleep diary was 81 (range: 26, 84). Subjective sleep efficiency is presented in Table 2.

**Table 1:** Characteristics of the participants.

Variables	Sleep onset insomnia Median (25th 75th quartile)	Non-sleep onset Median (25th 75th quartile)	Total (n = 20) Median (25th 75th quartile)
Age in years, mean SD	56 (53, 62)	57 (46, 57)	56.5 (51.5, 59.5)
Female, n	7	2	9 (45 %)
Male, n	4	7	11 (55 %)
PSG/SAS SE at baseline	85.2 (83.9, 96.5)	91.85 (88, 94.3)	90 (84.2, 96.5)
AHI at baseline	16.2 (11.1, 50.9)	24.25 (3.4, 58.8)	16.2 (8.75, 54.85)
Entries in the digital sleep diary	73 (26, 84)	84 (46, 84)	81 (26, 84)
Above median engagement	7	3	10 (52.63 %)
Below median engagement	7	2	9 (47.37 %)

Table presenting the demographic variables at baseline* for the two groups with median and range (25 and 75 percentile) since all variables were not normally distributed. Polysomnography/self-applied somnography (PSG/SAS). Apnoea hypopnea index (AHI). *Entries are post-study.

**Table 2:** Differences in subjective sleep parameters at baseline and post-study.

Variables	Baseline Median (25th, 75th percentile)	Post-study Median (25th, 75th percentile)
N	16	15
SSE	79.61 (74.41, 85.62)	89.47 (75.22, 91.9)
SSQ	2.92 (2.78, 3.14)	3.28 (2.71, 3.42)

The table shows the subjective sleep efficiency (SSE) and subjective sleep quality (SSQ) at baseline and post-study among all participants. The table shows the median and the range (25 % and 75 % percentile).

### 3.2 Qualitative results

In the interviews, six of the 20 participants said that their sleep had improved during the intervention period, while eleven participants found that their sleep was the same as before. However, among all the 20 participants, nine shared that the digital sleep diary and the self-help book helped them become more aware of specific lifestyle habits that affect sleep quality. Three participants stopped reporting in the digital sleep diary after eight days because the diary was too repetitive and boring. Another participant did not report at all because it made her stress more about her sleep difficulties.

In the thematic analysis, two main themes were identified: “structure and overview” and “usability and digital features”.

#### 3.2.1 Structure and overview

Upon reviewing the visualised provided statistics, several participants felt they got a better overview of their sleep behaviours. One of the participants illustrates this with the following:

I have gotten more structure in what I actually do and do not do … I look at the weekly statistics, and then see what I can improve to try to change some of the trends …. I think it has helped (referring to the digital sleep diary) because if I had not written it down, I would have followed in the same footsteps today. I love statistics, and I want to be in the upper half (referring to the weekly report of sleep efficiency). And it is probably a competitive instinct … …and the awareness that you get to see statistically that I am not that bad. (P9, our translation).

Almost all participants expressed that they had no overview of their sleep behaviours before participating in the study and using the sleep-diary in CAPABLE platform. Through the visualisations, some participants also managed to gain a better overview of their daily routines related to sleep. Some participants expressed that they reflected on this information. Another participant expressed the following:

I have probably been more aware of being more active during the day; I have been going out more, and also followed some training sessions during the week. And it has something to do with the fact that we are in this pandemic situation, so I sit in the home office all the time, and therefore, I have been a bit more careful that I get to move myself during the day (P3, our translation).

However, not all participants perceived the utility of the information provided:

To get a better overview, yes, but if it does help me, I’m not sure. (P10, our translation).

Another participant said:

No (referring to gaining an overview of sleep by using the digital sleep diary), I knew that I was awake at night. Now, I had to put a time perspective every time I was awake at night, but I was aware of this before using the digital sleep diary (P7, our translation).

Most participants said that they got a better overview of their sleep while using the digital sleep diary. However, some participants noted that it did not provide them a better overview because they already knew how their sleep was.

#### 3.2.2 Usability and digital features

The SMS reminder sent by the digital sleep diary was perceived as one of the most helpful features. Participants expressed that they would have forgotten to complete their entries without it. One participant expressed:

Yes, I got a reminder every day and it went well. If I had not received that reminder, I certainly would not have used it (referring to CAPABLE sleep diary) so well. Also, I was reminded about it every day, and then it was a habit of it every morning at 9 am. (P1, our translation).

Another participant said:

It is a must that you receive that (referring to the text message reminder). It was a period where you didn’t … where there was something wrong with it, and then it was like …, I have forgotten about it. So, that text message was important (P12, our translation).

Some participants suggested that other consumer technology could be utilised to add more objective data and assist in completing sleep diary entries. One participant said:

I imagine if you connected a watch or something like that to this (referring to digital sleep diary), you would have had the right sleep time and heart rate and all that (P9, our translation).

Several participants would like to see more information visualised, for example an overview of coffee, tea, and alcohol-intake and naptime during daytime. Further, some participants wanted to see more statistics and visualisations of their actual data and compare it to their expectations on how much sleep they should have. Also, some would have liked to compare their data with other users in the same age group, like one of the participant said:

It could be interesting because I could then see where I stand in relation to others, whether I am high above or in the middle or very low. It would have been interesting to follow along … (P5, our translation).

Beyond using CAPABLE platform for sleep diary, the participants were interested in incorporating other features and functionalities in the platform. Some of the mentioned features were related to sleep behaviour, such as pedometers and diets. Additional functionalities included communications with health personnel, list of medication and log of other medical conditions. One of the participant illustrates:

It might exist without me knowing, but something like registration of anxiety and symptoms of anxiety. And migraines. Because then it is about trying to find out what triggers the anxiety, what triggers the migraine, for example. … What happened when you had a bad day, because then it is about trying to figure out what is causing it. And what did you do that actually made it worse? So, I think anxiety and migraine registration can be useful with something like that. (P2, our translation).

Another participant said:

*I think it would be beneficial to get an overview of physical activity because it is connected to sleep.* (P15*, our translation*).

Many participants said that the daily reminder was crucial for their reporting in the digital sleep diary. Further, they seemed to like the functionalities available in the platform and above all, the simplicity of using the digital sleep diary. Still, several participants suggested other functionalities such as activity log, anxiety and migraine diary, and generally more interactive visualisations in the platform.

## 4 Discussion

We found an overall high engagement, with a median of 81 entries in the sleep diary per participant over the study period. However, the range of entries per person was large. The engagement suggest that using the sleep diary as implemented in the CAPABLE platform is feasible. This is also supported by a trend of more use in participants with sleep onset insomnia than in participants without. The semi-structured interviews identified primarily positive experiences with the use of the application.

Our sample's distribution of age, AHI and gender is comparable to other studies of co-morbid insomnia and obstructive sleep apnoea (COMISA) [6]. However, not all participants recruited for this study had COMISA. Moreover, we observed lower AHI in the sleep onset group than in non-sleep onset group. This observation is in line with a previous study that observed less severe OSA in sleep onset insomnia than in sleep maintenance insomnia [7]. Differences in these sub-types of insomnia should be explored in larger samples, in particular in patients with COMISA.

Our finding of a high number of entries could be related to the daily reminder to report in the digital sleep diary is in line with previous research [21,22]. This indicates that reminders can be an essential component in adherence to and the use of digital self-management tools. Time consumption and repetitiveness were reported as reasons for non-adherence to filling in the sleep-diary, which is also reported in previous studies [21,31]. Engagement was measured by the median number of entries in the digital sleep diary with a cut-off value of 81 entries. Surprisingly, the sleep onset insomnia group had lower engagement than the non-sleep onset insomnia group (73 vs 84 entries). However, the non-sleep onset insomnia group only consisted of five individuals.

Moreover, we found a trend of self-reported, improved sleep among the participants, in particular, in participants with sleep onset insomnia and high engagement. Related to these findings, other studies have reported that a sleep diary delivered on smartphones improves sleep efficiency when administered with other CBT-I components [19,20,22,32]. Moreover, five of the 11 participants who received the self-help book said that the book, in combination with the digital sleep diary, made them more aware of lifestyle factors that influence sleep. The awareness about lifestyle changes and the general sleep advice given in the self-help book, seemed to positively affect the participants. In a feasibility study of another mobile application called “CBT-I Coach”, which contained several features for insomnia treatment, reports personalised feedback from the sleep diary as helpful, and the most used component in the application [12]. This supports our finding that the graphical overviews and visualisations of the aggregated feedback in the CAPABLE sleep diary motivated and engaged participants to change lifestyle and behaviour to improve their sleep. This trend was seen in both groups.

Some participants suggested that the digital sleep diary in the CAPABLE platform would have been more beneficial if it had additional features such as an anxiety diary, overview of calorie intake and activity tracker. It was also suggested that the digital sleep diary could be connected to smartwatches. The literature supports these suggestions [19,20,22]. Several apps on the market have incorporated wearable technologies and sensors that can motivate and empower the user to take personal control of their lifestyle. This may provide opportunities for encouragement in their treatment and facilitate new ways to collaborate with clinicians to achieve patient-centred treatment [33,34]. On the contrary, using sensors may move focus away from self-rated sleep and importance of engagement, which is provided in current sleep diaries.

### 4.1 Limitations and strengths

This study has several limitations. The interviews were conducted seven months after the data collection, which could have resulted in the participants forgetting valuable information. Moreover, only participants in the clinical cohort underwent a sleep study before and after the intervention. Strengths of the study are that one author carried out all interviews and that traditional survey data and engagement were combined with the results of the qualitative interviews. The qualitative analysis was performed with three authors to ensure rigour and quality, and authors TCT, WKB, and AM reviewed the nodes, categories and themes. In this pilot study, we establish that the application is feasible and should be further tested in larger studies. In particular, the effect of the sleep diary should be tested in combination with digital delivery of CBTi elements such as sleep restriction and stimulus control.

## 5 Conclusion

The study shows that sleep diary was feasible when distributed in combination with a self-help book or EEG neurofeedback. The qualitative results emphasised the importance of the graphical overview and visualisations of self-reported sleep. Further, additional features to increase engagement were suggested. Future studies of the digital sleep diary should include more participants or more efficient additional interventions. Moreover, more research is needed to develop and test digital self-management tools and applications to support patients and clinicians with efficient, useful and safe tools. Digital tools like the digital sleep diary comes with this potential and can be a viable alternative to complement traditional CBTi consultations as it can be a cost-effective approach that is both time- and resource-saving as well as easily accessible for both patients and clinicians.

## Declaration of generative AI in scientific writing

**Statement**: After peer-review of this work the authors used [Grammarly] in order to improve the grammar and sentence structure. After using this tool, the authors reviewed and edited the content as needed and takes full responsibility for the content of the publication.

## Funding

This study was funded by Nord Forsk, NFR 298845. Development of the CAPABLE platform has been partly funded by 10.13039/501100005416Norwegian Research Council, project 282102 “CAPABLE – Empower Citizens to active use of their Health Information”.

## Declaration of competing interest

The authors declare the following financial interests/personal relationships which may be considered as potential competing interests: Harald Hrubos-Strom reports financial support was provided by Nordforsk.

## Abbreviations

ASAP II Epi: Akershus Sleep apnoea project II Epidemiological cohort
ASAP II clin: Akershus sleep apnoea project II Clinical cohort
OSA: obstructive sleep apnoea
CBTi: cognitive behavioural therapy for insomnia
AHI: apnoea-hypopnoea index
OSE: objective sleep efficiency
SSE: subjective sleep efficiency
SSQ: subjective sleep quality
EEG: electroencephalogram
SAS: self-applied somnography
PSG: polysomnography
P: participant
AASM: American Academy of Sleep Medicine
