# Supplementary material for: Experience with the use of a digital sleep diary in symptom management by individuals with insomnia -a pilot mixed method study
Source: Sleep Med X. 2023 Nov 14;6:100093. doi: 10.1016/j.sleepx.2023.100093 (PMC10757200; doi:10.1016/j.sleepx.2023.100093)
Supplement: Multimedia component 1 [file mmc1.docx]

**Supplemental Material**

**Sleep registrations**

Participants underwent a sleep registration. Either a Self-applied sleep registration or a Polysomnography (PSG). The sleep registrations was manually scored using the latest scoring rules by the AASM Manual for the scoring of sleep and associated events (version 2.6, 2020). The scoring was done by an expert sleep technologist at Reykjavik University Sleep Institute, using Noxturnal Research (version 6.1.0.30257).

Regarding scoring of SAS, the following are deviations from the AASM scoring rules, needed for the SAS EEG signal:

-The low pass filter setting for the EEG was changed from 0.3 Hz to 0.5 Hz. **

-The peak to peak amplitude of the slow wave activity was changed from 75 µV to 50 µV***. In the PSG it is measured over the frontal regions and referenced to the contralateral ear or mastoid (F4-M1, F3-M2). In the SAS it is referenced to the average of E4 and E3. The frontal filter available in the software was not used.

-The frontal EMG does not show loss of tone in REM in the same manner as chin EMG. This means that REM rules that refer to chin tone are not useable for the most part. But it is however possible use the EMG frontalis to aid in scoring arousals (in REM in particular). Arousals in REM were scored if there was an abrupt change in the EEG to faster frequencies accompanied by an increase in EMG tone as in the AASM scoring rules.

**Self-help book “Sleep well”**

A self-help book was given to the participants in the epidemiological cohort as an additional intervention when they participated in the ASAP II epidemiological study. The book describes what is characterized as good sleep and provides information on how to overcome sleep disorders, such as insomnia. The sleep advices given in this book is based on cognitive behavioural therapy for insomnia. Simple and practical relaxation exercises are also described to help initiate a good night sleep.

**Electroencephalogram neurofeedback device by Drowzee**

Participants in the clinical cohort received an EEG neurofeedback device when they participated in the ASAP II clinical study. The EEG neurofeedback device contains a mobile application providing automatized programs for sleep improvement (Drowzee AS) and a wearable brain-sensing headset developed by the Spanish company BitBrain. The intervention is based on EEG Neurofeedback. The neurofeedback algorithm in the application analyses the EEG signal and provides audio feedback when reaching specific patterns that Drowzee’s algorithm detect as sleepy brainwaves. Drowzee’s algorithm has been proven stable and consistent in company preclinical trials. The audio feedback will aim for teaching self-regulation of brain function and enhance the ability to reproduce the same patterns by the brain. For people with chronic insomnia or insomnia symptoms, this skill may be beneficial.

**Overview of the participants**

| Participant | Cohort | Sex | Age | Entries in the digital sleep diary | DUKE1a | DUKE1b | DUKE1c |
| --- | --- | --- | --- | --- | --- | --- | --- |
| 1 | 1 | 1 | 76 | 81 | 1 | 1 | 1 |
| 2 | 1 | 1 | 47 | 4 | 1 | 0 | 0 |
| 3 | 1 | 1 | 46 | 26 | 1 | 1 | 1 |
| 4 | 1 | 2 | 56 | 84 | 1 | 1 | 1 |
| 5 | 1 | 2 | 61 | 44 | 1 | 1 | 0 |
| 6 | 1 | 1 | 66 | 8 | 1 | 1 | 1 |
| 7 | 1 | 2 | 66 | 8 | 1 | 1 | 1 |
| 8 | 1 | 2 | 46 | 13 | 0 | 1 | 0 |
| 9 | 1 | 2 | 56 | 84 | 1 | 1 | 1 |
| 10* | 1 | 1 | 46 | - | 0 | 1 | 1 |
| 11 | 1 | 1 | 56 | 84 | 1 | 1 | 0 |
| 12 | 2 | 1 | 53 | 65 | 1 | 1 | 1 |
| 13 | 2 | 2 | 62 | 84 | 1 | 0 | 0 |
| 14 | 2 | 2 | 50 | 84 | 1 | 1 | 1 |
| 15 | 2 | 2 | 57 | 46 | 0 | 0 | 1 |
| 16 | 2 | 1 | 57 | 45 | 1 | 1 | 1 |
| 17 | 2 | 2 | 56 | 84 | 1 | 1 | 1 |
| 18 | 2 | 1 | 57 | 84 | 0 | 1 | 0 |
| 19 | 2 | 2 | 57 | 84 | 0 | 1 | 0 |
| 20 | 2 | 2 | 58 | 84 | 0 | 0 | 1 |

Cohort: 1= Epidemiological cohort, 2= Clinical cohort

Sex: 1 = Female, 2= Male

DUKE1a: Have you experienced difficulties falling asleep?

DUKE1b: Have you experienced difficulties maintaining sleep?

DUKE1c: Have you experienced waking up earlier than desired?

DUKE1a-c: 0=no, 1=yes

*Not included in the engagement variable
